# Resistance Patterns Associated with HCV NS5A Inhibitors Provide Limited Insight into Drug Binding

**DOI:** 10.3390/v6114227

**Published:** 2014-11-06

**Authors:** Moheshwarnath Issur, Matthias Götte

**Affiliations:** 1 Department of Microbiology and Immunology, McGill University, Montreal, QC H3A 2B4, Canada; E-Mail: moheshwarnath.issur@mail.mcgill.ca; 2 Department of Biochemistry, McGill University, 3655 Sir William Osler Promenade, Montreal, QC H3G 1Y6, Canada; 3 Department of Medicine, Division of Experimental Medicine, McGill University, 1110 Pine Avenue West, Montreal, QC H3A 1A3, Canada

**Keywords:** Daclatasvir, NS5A, HCV, resistance, resistance barrier, viral fitness, DAA

## Abstract

Direct-acting antivirals (DAAs) have significantly improved the treatment of infection with the hepatitis C virus. A promising class of novel antiviral agents targets the HCV NS5A protein. The high potency and broad genotypic coverage are favorable properties. NS5A inhibitors are currently assessed in advanced clinical trials in combination with viral polymerase inhibitors and/or viral protease inhibitors. However, the clinical use of NS5A inhibitors is also associated with new challenges. HCV variants with decreased susceptibility to these drugs can emerge and compromise therapy. In this review, we discuss resistance patterns in NS5A with focus prevalence and implications for inhibitor binding.

## 1. Introduction

The World Health Organization estimates that approximately 130–150 million people worldwide are chronically infected with the hepatitis C virus (HCV) [1]. A significant proportion of infected individuals develop severe liver disease, including cirrhosis and hepatocellular carcinoma (HCC) [2]. Since HCV related liver disease manifests itself after decades of infection, the risk of transmission is high and the global burden of hepatitis C is expected to increase despite the successful development and approval of novel treatments [3]. Anti-HCV therapy has remarkably evolved over the last decade. The necessity for novel drugs has been driven by poor response rates of interferon (IFN)- and ribavirin (RBV)-based treatments, especially in the context of infection with genotype 1. The first generation of direct acting antivirals (DAA) are represented by the inhibitors telaprevir and boceprevir that target the viral protease, *i.e*., nonstructural protein 3 (NS3) [4]. The two drugs were approved in 2011 for the treatment of genotype 1 chronic hepatitis C in combination with IFN and RBV. The second-generation protease inhibitor simeprevir, was approved in 2013 [5,6]. Sofosbuvir is a nucleotide analogue inhibitor of the viral RNA-dependent RNA polymerase (NS5B) and was also approved in 2013 [7,8]. It is expected that the numerous favorable properties of recently approved or investigational DAAs will pave the way for IFN/RBV-free treatment regimens.

Inhibitors of HCV NS5A represent yet another promising class of compounds [9]. Daclatasvir (DCV) is a prototype NS5A inhibitor that is currently being assessed in advanced clinical trials (Table 1). NS5A inhibitors show antiviral activity in the low picomolar range; however, the emergence of resistant HCV variants that decrease susceptibility to these drugs is of potential concern. NS5A inhibitors feature low barriers to the selection of resistance conferring mutations. For instance, a single nucleotide change in NS5A of HCV genotype 1a can lead to an amino acid change at position 93 that increases the effective concentration of DCV by several orders of magnitude.

## 2. Structure and Function of NS5A

HCV NS5A is an RNA-binding phosphoprotein [10,11]. Based on electrophoretic mobility it predominately exists in two forms: a hypophosphorylated (p. 56) and a hyperphosphorylated (p. 58) [12,13]. Reiss and colleagues have recently shown that the host factor, phosphatidyl-inositol-4 kinase III alpha (PIKIIIα) binds to and regulates the phosphorylation status of NS5A [14]. The phosphorylation state likely plays an important role in assigning its various roles in RNA replication, virus assembly, and packaging [12].

NS5A is anchored to the endoplasmic reticulum (ER) and ER-derived membranes through an N-terminal amphipathic α-helix. The structure for the membrane anchor has been solved by nuclear magnetic resonance spectrometry (NMR) for genotype 1a [15]. It is thought that this structure is conserved across all HCV genotypes. NS5A is further subdivided into three domains and two linker regions [16]. The structure of Domain I from genotype 1a and 1b has been solved by X-ray crystallography [17,18,19]. All three crystallographic studies reveal a similar fold of NS5A Domain I around a zinc atom coordination architecture that involves four cysteine residues. Despite the structural resemblance at the tertiary level, each structure shows unique homodimeric conformations. The existence of an RNA binding channel is plausible at the dimer interface of one of the structures [19]. However, the functional relevance of each of the three conformations remains to be elucidated. Domains II and III of NS5A are essentially unstructured and highly flexible [20,21]. It is this flexibility that may explain the broad spectrum of functions that have been associated with NS5A. Often termed a “promiscuous” protein, NS5A interacts with a wide array of cellular and viral factors [22,23,24]. Viral factors include the HCV NS5B and the viral RNA [25]. Cellular factors include various kinases as well as the lipid membrane of the ER that form pockets of HCV replication within “membranous web-like” structures. Domain II of NS5A interacts with the host factor cyclophilin A (CypA). Disrupting this interaction is detrimental to viral replication, and several CypA inhibitors are potent antivirals [26]. Of note, mutations that emerge under selective pressure of CypA inhibitors are located in domain II of HCV NS5A.

## 3. Possible Mechanisms of NS5A Inhibitors

DCV, the first in class inhibitor, displays very high potency in cell culture assays with effective concentrations between 4–20 pM [9]. Although the target of DCV was initially unknown, drug resistance conferring mutations were mapped to NS5A. Domain I of NS5A was shown to be the most likely target of DCV when it was subsequently demonstrated that DCV directly binds with high affinity to this region of NS5A [27,28]. Inhibitor binding was shown to downregulate the hyperphosphorylation of NS5A [29]. This would lead to several downstream events, which include unusual protein localization, inhibition of poly-protein processing and termination of HCV replication. Initially, HCV genome replication has been the major focus with respect to possible mechanisms of action of these inhibitors. Several studies have aimed to precisely identify the specific effect of DCV on the replication complex. More recent studies are, however, showing that the mechanism of DCV might not be restricted to its effects on viral replication. A new study conclusively showed that DCV derivatives completely blocked the formation of the membranous web structure, into which genome replication occurs [30]. Such an effect would explain the very high potency of NS5A inhibitors. Blocking the biogenesis of the membranous web would represent a novel paradigm in antiviral therapy. In addition, recent kinetic studies by McGivern and colleagues have shown that NS5A inhibitors target viral assembly at the onset of inhibition [31]. Their effect on RNA replication was relatively slow. These findings correlate with previous clinical data revealing a multiphasic decline in serum HCV following treatment with DCV [32,33]. An initial rapid decline (t_1/2_ = 48 min) of HCV followed by a slower decline phase (t_1/2_ = 6–9 h) was observed. The initial decline phase was attributed to inhibition of the viral assembly or secretion pathway, and the second phase to genome replication. Overall, both clinical and *in vitro* data support a dual mechanism whereby both viral RNA replication and viral assembly are targets of NS5A inhibitors.

## 4. Resistance to HCV NS5A Inhibitors *in vitro* and *in vivo*

**Figure 1:**
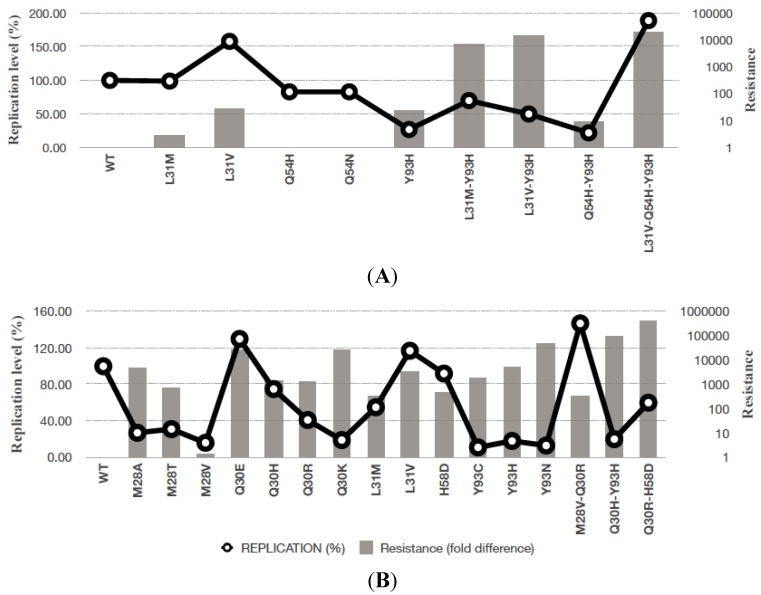
Fitness and level of resistance of DCV-associated mutations assessed against HCV genotype 1a (**A**) and 1b (**B**) replicons. The bar chart represents the fold decrease in drug susceptibility of HCV variants compared with WT. Open circles show differences in replication capacity, respectively. The figure is adapted from Fridell and colleagues [34].

Mutations associated with failure of DCV mono- or combination-therapy are often identical to those selected in the HCV replicon system or with the infectious clone [34]. Resistance patterns derived from clinical samples can, however, differ in complexity [35]. Adaptive and linked substitutions, which do not directly contribute to resistance, could co-emerge. A summary of DCV resistance associated mutations and their effect on HCV replication *in vitro* is given in Figure 1 [34,36,37]. Most of these amino acid substitutions map to the N-terminus of NS5A (Figure 3). The primary resistance conferring mutations for genotype 1a are M28T, Q30E/H/R, L31V/M, P32L and Y93H/N and for genotype 1b are L31V/F, P32L and Y93H/N. Mutations at Q30, L31 and Y93 confer the highest levels of resistance in both subtypes. Genotype 1a shows generally lower barrier to the development of resistance when compared to genotype 1b [9]. The high potency of NS5A inhibitors implies that perhaps multiple mutations are required to confer significant levels of resistance. The genotype 1b L31V and Y93H mutations individually confer 24- and 28-fold resistance to DCV respectively, which increases to approximately 15,000-fold for the L31V-Y93H double mutation. The synergistic effect points to complementary, interlinked mechanisms. This pattern also leads to decreased susceptibility to DCV in the other HCV genotypes. Although these variants show cross-resistance to the other NS5A inhibitors, they remain fully sensitive to other DAA classes and host targeting antivirals [34,38,39], including CypA inhibitors Alisporivir and SCY-635 [40,41].

## 5. Effect of Genotypic Variation on the Efficacy of NS5A Inhibitors

In the prior DAA era, the prognosis for patients with HCV genotypes 2 and 3 was much better than for patients infected with genotypes 1 and 4 [42,43]. The addition of NS3 protease inhibitors to the previous standard of care has greatly improved SVR rates in genotype 1 patients [44,45]. NS5A inhibitors display favorable efficacy across all major genotypes and are likely to be a component of any multi-drug regimens with pan-genotypic activity. However, pre-existing, genotype specific polymorphisms may still affect response to treatment [46].

**Figure 2:**
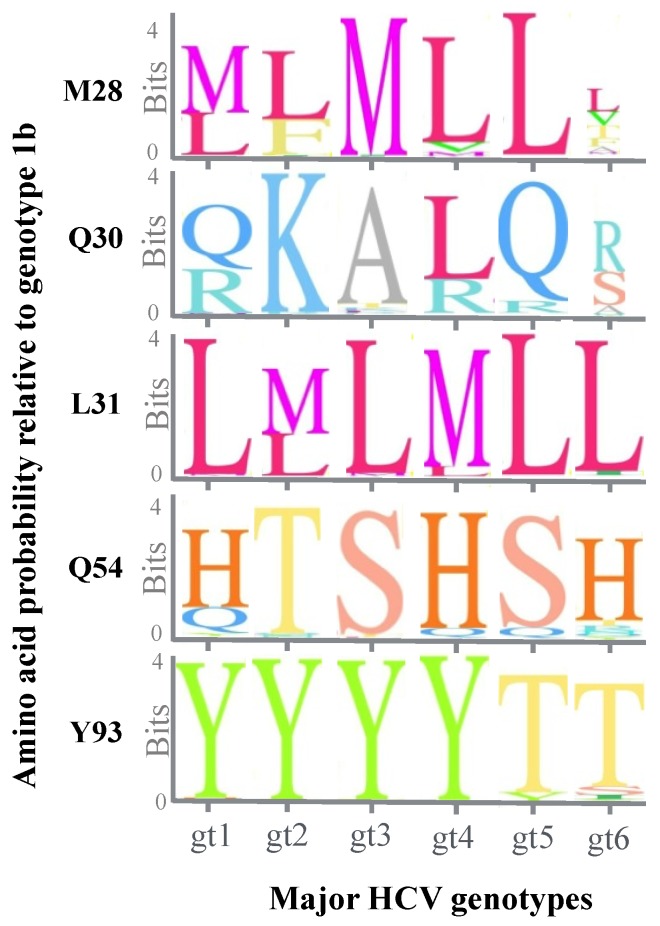
Sequence logo diagram showing sequence heterogeneities at sites associated with DCV resistance. Sequences from each major genotype from the Los Alamos HCV database was aligned using the geneious software [51]. The height of letters representing relevant amino acids indicates their probability at that position.

Residues 30, 31 and 93 are the major sites associated with resistance to DCV in most of the genotypes [47,48]. In genotype 3a, linked substitutions at L31 and Y93 are detected under DCV selection pressure [49]. Similarly, in genotype 4, residues 30, 31 and 93 are the major resistance conferring mutation sites [50]. Linked substitutions L30I-Y93R confer approximately 9000-fold resistance to DCV. In genotype 5a and 6, several of the resistance-conferring sites are not conserved (Figure 2). In genotype 5a, major mutations are L31V/F [47]. The secondary K56R mutation is also selected and enhances six–10-fold the pre-existing resistance levels of L31V/F. The P32L mutation confers the highest level of resistance for genotype 6. L31M, P32S and T58A/N/S are also selected as DCV-resistance conferring mutations. The change in resistance patterns between genotypes 1–4 and genotypes 5–6 highlights heterogeneity within primary structures across the major HCV genotypes.

An alignment of sequences from the Los Alamos HCV database to NS5A from genotype 1b also reveals sequence heterogeneity at several resistance-associated sites (Figure 2). For instance, the amino acid at position 30 is not highly conserved and the Q30R substitution pre-exists in genotypes 1, 4 and 5. Positions Q30 and Q54 are highly variable across the other genotypes. Y93 is highly conserved in genotypes 1–4 but not in 5 and 6. The facile selection of drug resistance has driven the development of a new generation of NS5A inhibitors, which promise to retain their potency against variants resistant to the “first generation” of NS5A inhibitors. Previous structure-activity relationship (SAR) studies revealed that increased rigidity of the bi-phenylic core of the NS5A inhibitor contributes to inhibitor potency [52]. SAR studies and selection against resistant HCV variants led to the development of MK-8742. MK‑8742, ACH-3102 and GS-5816 are such second-generation NS5A inhibitors currently in clinical trials (Table 1). Interestingly, the symmetry of the initial NS5A inhibitors was not conserved in these compounds. These new inhibitors retain sub-nanomolar potency against major, previously described variants. This includes variants featuring substitutions at positions 31 and 93. In genotype 1a, single amino acid changes within the NS5A region leads to very high levels of resistance to DCV, while with MK-8742 the fold resistance is substantially lower [52]. No data is available on the potency of these drugs in the case of linked mutations at positions 31 and 93.

**Figure 3:**
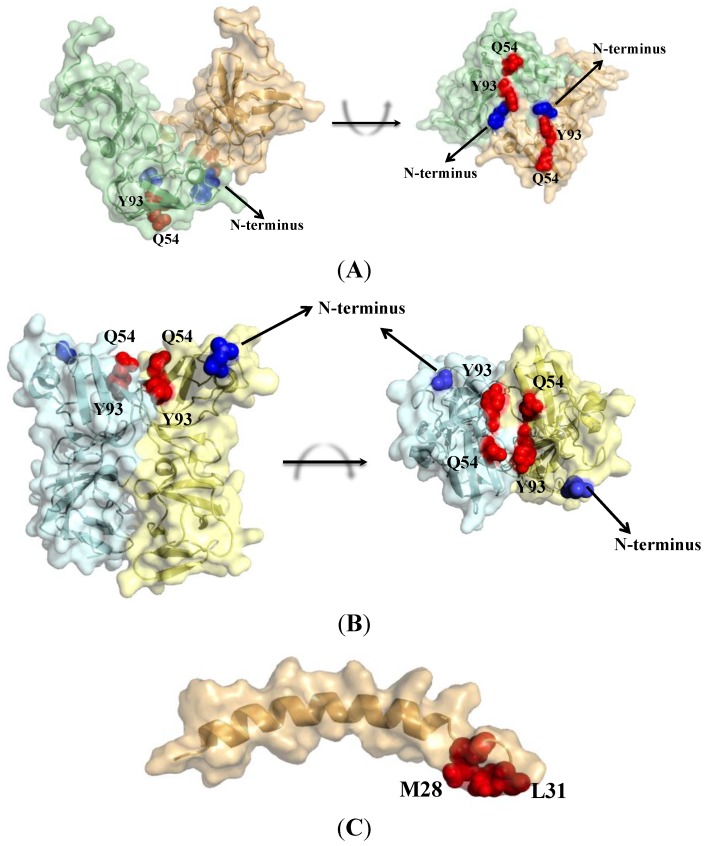
Structures of NS5A domain I. Amino acid positions associated with resistance to DCV are shown as red spheres. The N-terminal amino acid in each monomer is shown in blue. (**A**) Front view and a 90° in plane rotation of the “open” conformation of NS5A (36–198) (PDB 1ZH1); (**B**) The side view and an out-of-plane rotation of the “closed” conformation of NS5A (32–191) (PDB 3FQQ); (**C**) The structure of the N-terminal amphipathic helix of NS5A (1–31) (PDB 1R7G). It is expected to link with each monomeric structure at the N‑terminal amino acid.

## 6. Resistance Mutations Define Binding

For NS5A inhibitors, the emergence of resistance has played an important role in defining the target of inhibition. Resistance conferring mutations at position 31 and 93 in domain I of NS5A and pronounced synergistic effects point to a high affinity-binding site for the NS5A inhibitors. The symmetric nature of first generation NS5A inhibitors and the fact that DCV pulls down higher order structures suggests that these compounds target dimers. A systematic functional analysis of several DCV related compounds lead O’Boyle and colleagues to propose that the potent antiviral activity combines distinct pharmacophores across the NS5A dimer interface [27]. Taken together, resistance data and the symmetry of most NS5A inhibitors around a common conjugated aromatic core indicate binding to a dimer of NS5A, which will conserve symmetry at the inhibitor-binding site. In this regard, crystallographic structures of NS5A Domain I of genotype 1b are useful in inferring binding (Figure 3) [53]. In the recently published crystal structures of Domain I of NS5A from genotype 1a, resistance conferring mutations do not reside within a symmetrical interface [17]. However, it is worth mentioning that Lambert and colleagues do propose a mode of binding of DCV to one of the crystalized conformation. However, the resistance conferring Y93 site is markedly distant from the aromatic core of DCV. In addition, the Y93 resistant conferring site is shielded from direct interaction with DCV. The authors argue for an allosteric mode of resistance to DCV. However, the current favored model of resistance involves direct binding. Therefore, the crystal structures from NS5A of genotype 1b will be mainly discussed in relation to DCV binding.

The first crystal structure of NS5A encompasses amino acids 36–198. It features a deep groove between the monomeric sub-units and is speculated to be able to accommodate an RNA strand [19]. This structure will be called the “open” conformation. The second structure from Love and colleagues is a “back-to-back” dimer structure and will be called “closed” [18]. The resistance conferring mutations in all HCV genotypes are clustered at the dimer interface in a region proximal to the membranous anchor of NS5A- the amphipathic helix (Figure 3). In the “closed” conformation, these mutations lie around a shallow cleft at the membrane-proximal region of the dimer. This cleft is absent in the hypothetical “open” RNA binding conformation. However, in both conformations, the resistant mutations depict an inhibitor-binding interface, which spans across both units of the dimer (Figure 3).

Molecular docking studies have further refined this model. Since available crystal structures exclude the amphipathic helix and the associated linker region, it has to be either modeled into the structures or completely excluded from the molecular docking procedures. Both scenarios indicate that DCV can bind to both structures. Docking indicates that the bi-phenylic core of DCV stacks with Y93 from each subunit at the binding site [53]. This interaction is stabilized by hydrogen bonds between the rest of the molecule and R30 and Q54. A secondary contact site involves position 58, to which secondary resistance conferring mutations have been associated with. Through the use of state of the art docking procedures, Bharakat and colleagues recently proposed a pharmacophore modeling for the binding of DCV to NS5A. This model relates satisfactorily with existing resistance data. Qi and colleagues recently established a drug resistance and sensitivity profile for the whole DCV binding region by a saturation mutagenesis approach [54]. Novel resistance conferring mutations were identified. Residues 28, 31, 38, 92 and 93 contributed to resistance while residues 21, 56 and 58 increased sensitivity towards DCV in a chimeric J6/JFH1 (genotype 2a) virus. Importantly, the nature of the amino acid substitution at positions 24, 30, 62 and 75 were determinant for either increased susceptibility or resistance to DCV. However, molecular docking simulations have limited their binding studies to symmetrical binding interfaces. Since, symmetry of the binding pocket appears to be an important consideration for DCV binding, we presume that an asymmetric binding site involving a heterodimer between a wild-type and a resistant NS5A variant would enhance the observed reduced binding to DCV in the simulations involving homodimeric NS5A resistant variants.

## 7. Barriers to the Selection of Drug Resistance

The genetic barrier, the level of resistance gained, and the fitness of a given variant are important parameters that determine the outcome of a selection process [55]. The fidelity of the HCV polymerase directly affects the genetic barrier [56]. The HCV polymerase favors incorporation of G:U and U:G mismatches, which translates in a bias towards transition mutations over transversions. During a 14-day monotherapeutic clinical trial with DCV, the high resistance acquisition site, Y93, is initially selected for Cys (UAC to UGC) or His (UAC to CAC) substitutions, both of which are transitions. Selection of the highest drug-resistance conferring mutation, Y93N (UAC to AAC), through transversions, occurs rarely [34]. However, the Y93H/C variant is severely compromised in replication capacity and is rapidly lost unless it is linked with substitutions at other residues that neutralize the fitness deficit. Transversions are sometimes selected if equivalent transition mutations show greater costs in viral fitness. For example, even if resistance-conferring mutations P32L (CCG to CUG) and C92R (UGU to CGU) occur via transitions, these mutations are rare. Instead L31M/V substitutions, which occur via transversions (UUG to AUG and UUG to GUG respectively), are commonly selected.

## 8. Conclusions

A potential limitation to the clinical use of NS5A inhibitors is a relatively low barrier to the development of resistance. Selection kinetics vary according to the HCV genotype [47]. Genotype 1a seems to be prone to the generation of single amino acid changes in the N-terminal portion of NS5A that lead to high levels of resistance. However, the use of NS5A inhibitors in combination with other DAAs greatly reduces the risk of resistance selection [57,58]. Combinations with either NS3 protease or NS5B polymerase inhibitors show promising results in this regard. Clinical trials of treatment regimens involving DCV, Asunaprevir and the polymerase inhibitor, BMS-791325 displayed very promising results in untreated genotype 1a and 1b subjects for an all-oral, IFN/Ribavirin free treatment option [57]. Although second generation NS5A inhibitors are already in development, the precise binding mode and mechanism of action remain to be ascertained. Several models, involving different conformations of NS5A, have been proposed. *In vitro* studies indicate a 25-fold difference in affinity when DCV binding is assessed against the two different conformations of NS5A [28]. This implies that the inhibitor may bind to both conformations at physiological conditions, albeit to different degrees. Further studies into the effects of resistance conferring mutations on the conformational flexibility of NS5A may help to provide a more detailed understanding associated with drug binding and mechanism of action.

**Table 1:** HCV NS5A inhibitors currently in clinical trials.

NS5A Inhibitor	Clinical Trial	Drug Combination	Status
Daclatasvir (BMS-790052)	Phase III completed	+Asunaprevir	Submitted for FDA approval [59,60]
		+polymerase inhibitor (Sofosbuvir or VX-135) ± Protease inhibitor ± Ribavirin	Genotypes 1, 3, 4 Under investigation for use in HCV-HIV co-infected cohorts [61,62,63,64,65]
Ledipasvir (GS-5885)	Phase III completed	+Sofosbuvir	Approved by FDA for genotype 1 [66,67]
GS-5816	Phase III	+Sofosbuvir	Genotypes 1,2,3,4,5,6 [68,69]
ACH-3102	Phase II completed	+Sofosbuvir	Genotype 1 (≤8 weeks treatment) [70]
Samatasvir (IDX-719)	Phase II	+Simeprevir (protease inhibitor) ± TMC647055 (polymerase inhibitor)	Genotype 1, 4, 6 [71]
GSK2336805	Phase II	+ Simeprevir + PEG-IFN + Ribavirin	Genotype 1 or 4 [72]
PPI-668	Phase II	+ Faldeprevir (protease inhibitor) + B1207127 (polymerase inhibitor)	[73]
PPI-461	Phase Ib completed	-	Genotype 1 [74]
TD-6450	Phase I	-	Potent against genotype 1a [75,76]
JNJ-47910382	Phase I	-	Asian genotype-1 [77]
